# Difficulties Encountered While Using PPE Kits and How to Overcome Them: An Indian Perspective

**DOI:** 10.7759/cureus.11652

**Published:** 2020-11-23

**Authors:** Ankur Agarwal, Sheetal Agarwal, Poonam Motiani

**Affiliations:** 1 Orthopaedics, Super Specialty Paediatric Hospital and Post Graduate Teaching Institute, Noida, IND; 2 Pediatrics, Atal Bihari Vajpayee Institute of Medical Sciences & Dr. Ram Manohar Lohia Hospital, New Delhi, IND; 3 Anaesthesiology, Super Speciality Paediatric Hospital and Post Graduate Teaching Institute, Noida, IND

**Keywords:** covid-19, sars-cov2, novel corona virus, ppe, personal protective equipment, difficulties, challenges.

## Abstract

Background

After a slow start due to an effective lockdown, the coronavirus disease 2019 (COVID-19) pandemic in India has been raging at a rapid pace, posing a formidable challenge to the healthcare system in the country. The personal protective equipment (PPE) undoubtedly provides a shield of protection for the healthcare workers (HCWs) fighting the disease as a valuable asset to the nation. However, there have been various problems associated with the PPE, ranging from its shortage to problems arising from heat, dehydration, etc while wearing them. There is a need to assess these problems faced by HCWs both qualitatively and quantitatively for their timely and effective redressal.

Methods

An electronic questionnaire survey was conducted among a cohort of HCWs who had performed COVID-19 duties and used PPE kits. The cohort consisted of different categories of doctors, nursing personnel, and other paramedical staff.

Results

The most common problems associated with using PPE kits was excessive sweating (100%), fogging of goggles, spectacles, or face shields (88%), suffocation (83%), breathlessness (61%), fatigue (75%), headache due to prolonged use (28%), and pressure marks on the skin at one or more areas on repeated use (19%). Occasional problems reported were skin allergy/dermatitis caused by the synthetic material of the PPE kit, face shield impinging onto the neck during intubation, and nasal pain, pain at the root of the pinna, and slipperiness of shoe covers. Various ways and means have been employed by the HCWs to actively address and solve these problems.

Conclusion

These plausible solutions will definitely help the HCWs to deal with and solve the problems arising out of the PPE use.

## Introduction

The coronavirus disease 2019 (COVID-19) pandemic has emerged as a major healthcare challenge worldwide. With over 4.8 million cases, India is the second-worst affected country so far. Due to India’s high population base, the highest daily incidence rate, and one of the highest daily mortality rates in the world, containing the spread within the country has turned out to be a Herculean task [1]. The first documented occupational transmission of COVID-19 among healthcare workers (HCWs) outside China occurred in California in February 2020. At this early stage, personal protective equipment (PPE)-related precautions were not well known. Consequently, out of the 121 HCWs who were exposed to COVID, 43 (36%) became symptomatic [2]. Official reports in India released to the media have claimed that more than 87,000 HCWs have already been infected with the disease and more than 573 COVID-related deaths have already occurred among HCWs till September 10, 2020. Hence, keeping the COVID-19 workforce safe has presented a daunting challenge [3]. From being used by beekeepers as reported in ancient literature, to 16th-century plague doctors in Europe to modern times, PPE kits have come a long way [4]. They form a very important part of the protective armour for the frontline warriors in this battle against the COVID-19 pandemic [5]. It is important to carefully select the adequate PPE to protect the skin, eyes, face, nose, mouth, hands, feet, head, and other parts of the body, so as to provide protection and act as an effective barrier between the HCW and the contaminated materials like blood, body fluids, respiratory secretions, and aerosols. The PPE usually comprises protective clothing, helmets, goggles, shoe covers, and respiratory protective equipment (RPE) [6]. Proper instructions, training, and supervision are required to ensure that the PPE is properly used and adequated protection is gained.

With the emergence of this unique challenge faced by modern medicine worldwide, the word PPE has been trending on Google Search engine [7]. Globally, the users have often found wearing the PPE uncomfortable while working, more so in the summer season, when facilities for controlling the environmental temperature like centralised air conditioners are unavailable or are shut down for fear of spreading the infection. In addition to reduced tactile sensitivity and impaired visibility due to the deposition of water vapours on the eye goggles with their use, users have also found verbal communication difficult while wearing the PPE. Although the literature has started to address and highlight the problems and issues related to PPE use on a global scale, there is still a dearth of authentic literature pertaining to the issue from within India.

Hence, we believe there is a need to evaluate and have a qualitative and quantitative assessment of the problems faced by HCWs in their use of PPE in India. The outlined aim of the study was to identify the difficulties encountered by HCWs while using PPE kits and to propose ways and means to help them overcome these difficulties.

## Materials and methods

A descriptive study was conducted in July 2020, by a team of researchers working in a government-owned super-specialty institute in North India, which was a designated COVID-19 treatment centre. An electronic questionnaire was prepared by the researchers for a multi-centre survey in urban India among HCWs who had used PPE kits during their COVID duties (Table 1). Internal consistency of the questionnaire was validated by keeping a spectrum of discrete options, eliminating the scoring system, and having a simple multiple-choice format. Content validation and construct validation of the questionnaire had been done by independent assessment by the two investigators in different time scales. Inter-rater reliability was ensured by allowing the respondents to fill in the questionnaires by themselves and keeping the questions simple. Test-retest validation of the questionnaire was ensured by allowing the respondent to edit the responses even after the first submission. During questionnaire validation, the average response time was also noted down.

**Table 1:** An overview of the questionnaire COVID: coronavirus disease; PPE: personal protective equipment; CPR: cardiopulmonary resuscitation

Questionnaire
Which hospital area have you done your COVID duty in?
How many days of COVID duty have been done by you?
What have been your daily average of duty hours?
How many PPE kits do you normally use per duty?
Did you have free availability of PPE kits?
What is the approximate duration of wearing one PPE kit?
What components of PPE kits/protective gear did you normally use?
Do you face any size problem with the PPE kit or any of its components?
Did you ever have any problem with the PPE kit getting torn at one/more places?
Did you face any problem in patients recognizing you due to the PPE kit?
Did you have any communication problems with staff/colleagues/patients during the PPE kit use?
Did you face difficulties in doing intubation with the PPE kit on?
Did you face difficulties in doing CPR with the PPE kit on?
Which other issues did you face while using the PPE kit?
Were you ever forced to remove the PPE kit due to severe thirst or dehydration?
Were you ever forced to remove the PPE Kit due to the urge for voiding the bladder?
Were you given prior training in donning/doffing of the PPE kit?
Did you have a separate designated area for donning and doffing?
Was someone available to help you in donning and doffing?
What steps did you take to overcome the problems you faced?

There was no absolute set of rules available to calculate the target sample size for this questionnaire, and an estimation could not be done as there was no previous study of a similar kind available in the literature. The respondent-to-item ratio principle was used to estimate the effective sample size. The number of compulsory responses in the questionnaire was 20. Hence, as per the 5:1 ratio rule for sampling size, a minimum of 100 respondents was required. After deliberation, the Institutional Ethics Committee granted permission to recruit a maximum of 300 subjects for the survey (IEC number: 2019-18-IM-02). The invitation to participate in the study was sent through email to HCWs who had done COVID duties and worn PPE kits at various government-designated COVID hospitals. The PPE kit, in our study, was defined as a hazmat suit and its accessories, including the N95 mask. The inclusion criteria were as follows: HCWs who had completed at least six days of COVID duty and worn PPE kit during each duty at least once. Those accepted to be part of the survey were sent a link to the questionnaire, either through email or WhatsApp mobile application (Facebook, Inc., Menlo Park, CA). The submission form was kept open for a period of 30 days.

Outcome measures were recorded automatically upon submission to Google Drive (Alphabet Inc., Mountain View, CA) and downloadable in the form of Microsoft Excel (Microsoft Corporation, Redmond, WA) spreadsheet tabulation. During the first stage, de-duplication of data was done using the unique mobile number fields in the Excel sheet. In the next stage, the validation of data was done, followed by data categorisation, preliminary analysis, and graphical representation. Subsequently, the result interpretation was done by the application of statistical analysis using SPSS Statistics software version 16.0 (IBM, Armonk, NY).

## Results

**Figure 1:**
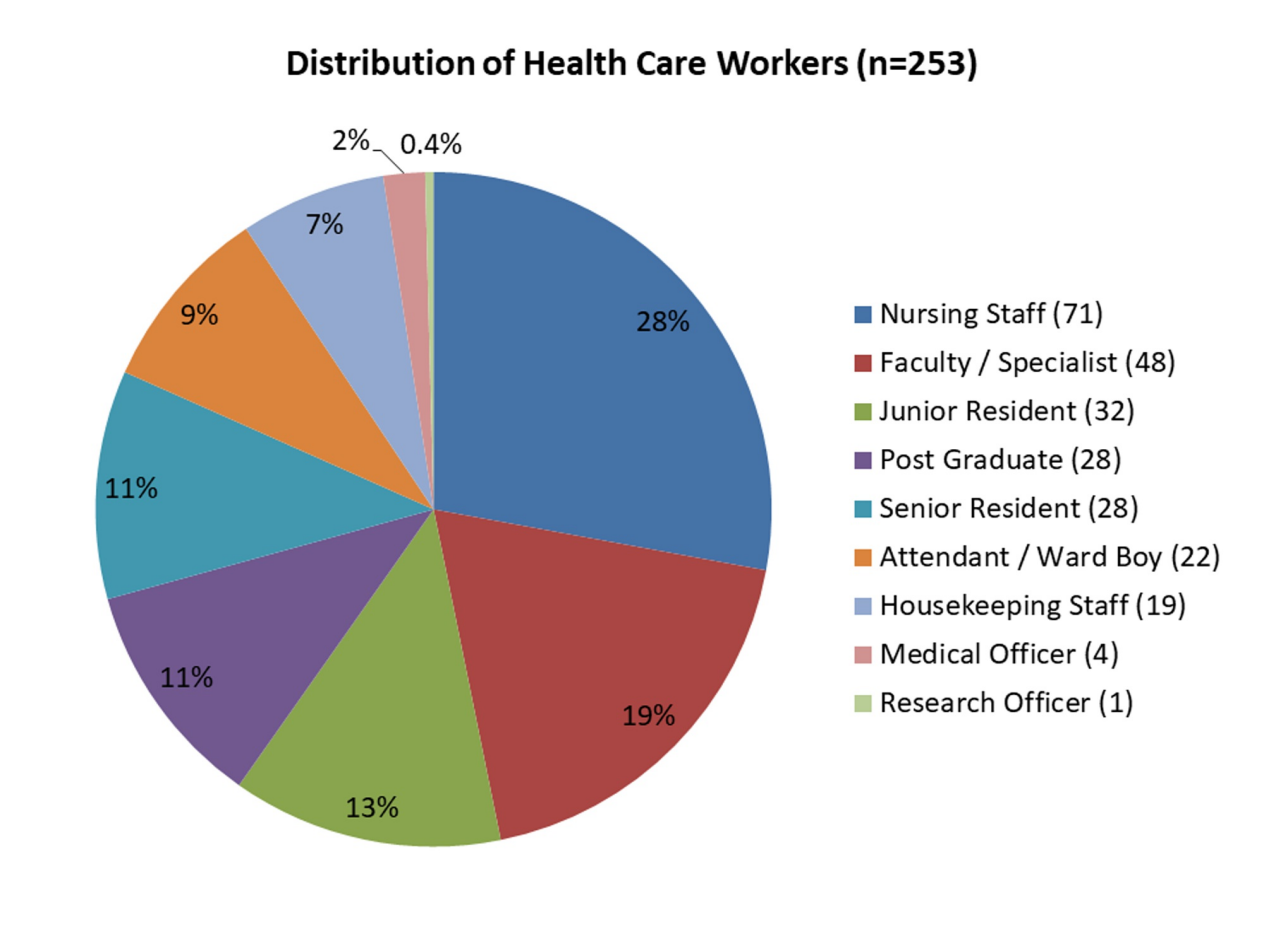
Detailed distribution of healthcare workers

We contacted 300 HCWs to be part of the survey, out of which 47 declined to participate. In total, 278 form responses were received at the close of the study. After de-duplication, 253 unique response forms were validated. The respondents included 140 doctors (55%), 72 nursing personnel and other technical staff (28%), and 41 ancillary staff (17%) from 26 different medical institutions, actively involved in COVID care. The detailed distribution of HCWs is depicted in Figure 1. Forty-six subjects (18.2%) declined to disclose their associated institution; 200 respondents had done their duty in COVID/suspect ward, 70 in ICU settings, 38 in screening area or sample collection centre, 20 in COVID/suspect operation theatre, and one in reverse transcription-polymerase chain reaction (RT-PCR) lab.

We found that 101 (40%) respondents had completed more than 28 days of COVID duty, 83 (33%) had done 7-14 days, 49 (19%) had done 15-28 days, while 20 HCWs (8%) had done up to seven days of COVID duty at the time of the survey submission. The median completed duty period was 15-28 days (IQR=7-14 days). As for hours/day of duty, 131 respondents (52%) had done 8-12 hours of duty per day, 48 (19%) had done 12 hours or more, 44 (17%) had done six to eight hours, while 30 (12%) had done up to six hours of COVID duty per day.

The survey revealed that 104 respondents (41%) had used a single PPE kit per shift, 91 (36%) had used mostly two PPE kits, 37 (15%) reported using mostly three PPE kits, 20 (8%) reported using around four PPE kits, and one respondent (0.4%) reported using more than four PPE kits in most shifts. The median PPE kit usage was two (IQR=1); 92 respondents (36%) reported wearing each PPE kit for approximately four hours, 58 respondents (23%) reported wearing it for approximately more than six hours, 44 respondents (17%) reported wearing it for approximately three hours, 32 respondents (13%) reported wearing it for approximately five hours, 25 respondents (10%) reported wearing it for approximately two hours, and two respondents (1%) respondents reported wearing each PPE kit for approximately one hour. The median duration was four hours (IQR=2) (Table 2).

**Table 2:** Distribution of the number of PPE kits used per duty among various classes of HCWs HCW: healthcare workers; IQR: interquartile range; PPE: personal protective equipment

PPE used (per duty)	Doctors, n=140, n (%)	Nursing personnel, n=72, n (%)	Ancillary staff, n=41, n (%)	Total, n=253, n (%)	P-value
1	75 (54)	22 (31)	7 (17)	104 (41)	<0.001(s)
2	42 (30)	39 (54)	10 (24)	91 (36)	
3	16 (11)	10 (14)	11 (27)	37 (15)	
4 or more	7 (5)	1 (1)	13 (32)	21 (8)	
Correlation-p	0.334				-
Median (IQR)	2 (1)				-
Kruskal-Wallis test (mean ranks)	109.73	130.39	180.01	-	<0.001(s)

The proportional usage of the number of PPE kits per duty was highest among ancillary staff, followed by nursing personnel and doctors. This is probably reflective of the work profile of each category (Table 2). Also, the nursing personnel wore PPE kits for a longer duration compared to other classes of HCWs, but this correlation did not come out to be significant with respect to various categories of HCWs (Table 3). The post-hoc comparison of problems of wearing PPE kits with respect to the approximate duration of use of each kit did not come out to be significant for any of the parameters.

**Table 3:** Comparison of duration of wearing of each PPE kit among various classes of HCWs HCW: healthcare workers; IQR: interquartile range; PPE: personal protective equipment

Each PPE duration	Doctors, n=140, n (%)	Nursing personnel, n=72, n (%)	Ancillary staff, n=41, n (%)	Total, n=253, n (%)	P-value
1 hour	2 (1)	0 (0)	0 (0)	2 (1)	0.002(s)
2 hours	17 (12)	2 (3)	6 (15)	25 (10)	
3 hours	26 (19)	8 (11)	10 (24)	44 (17)	
4 hours	50 (36)	25 (35)	17 (42)	92 (36)	
5 hours	10 (7)	19 (26)	3 (7)	32 (13)	
6 hours or more	35 (25)	18 (25)	5 (12)	58 (23)	
Correlation-p	0.018				-
Median (IQR)	4 (2)				-
Kruskal-Wallis test (mean ranks)	121.95	150.21	103.5	-	0.001(s)

## Discussion

There has been a flurry of research in recent literature on coronavirus and COVID-19, centering on its epidemiology, etiopathogenesis, pathology, prevention strategy, components of prevention, and treatment. However, none of the studies from within India has assessed the difficulties encountered by HCWs while using PPEs.

**Figure 2:**
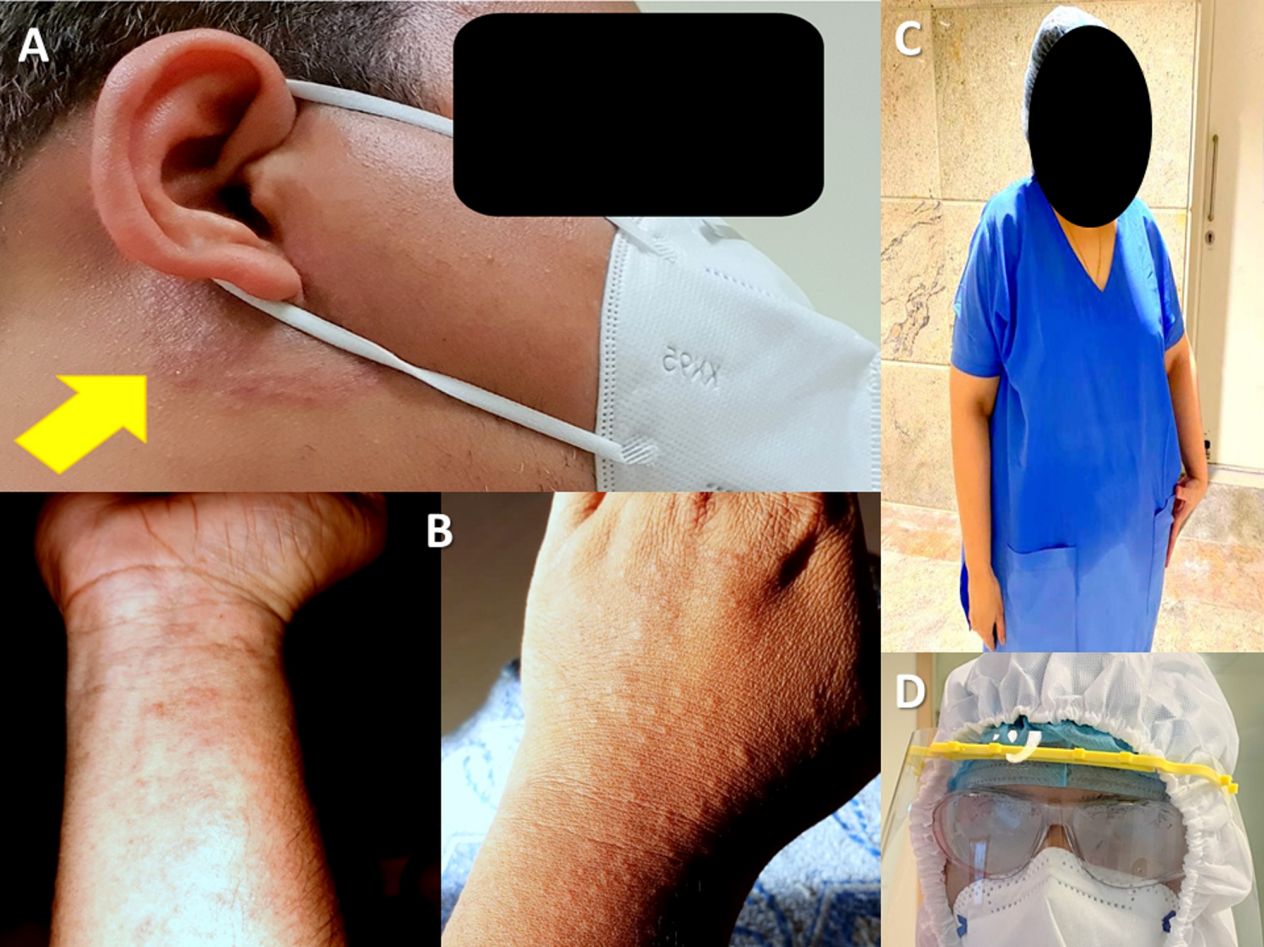
Depiction of a few problems faced while wearing PPE A: dermatitis; B: rashes; C: drenching in sweat; D: fogging PPE: personal protective equipment

In our study, the most common problems associated with using PPE kits were excessive sweating (100%), fogging of goggles, spectacles, or face shields (88%) (Figure 2), suffocation (83%), breathlessness (61%), fatigue (75%), headache due to prolonged use (28%), and pressure marks on the skin at one or more areas on repeated use (19%). India is a tropical country with hot and, at times, both hot and humid conditions. Hence, this problem was even more daunting. Shutting down central air conditioning systems (with common air duct systems) in the hospitals to prevent the spread of droplets and droplet nuclei further aggravated this problem. Features of dehydration like muscle cramps, dizziness, vertigo, and nausea were also reported on continuous use. We had a report of one respondent actually collapsing due to symptoms similar to heatstroke and had to be hospitalised. Similar reports are not uncommon from other centres [8]. Respondents reported drinking moderate quantities of cool water before donning (74%), frequent change of kits with intervals in between (59%), and using AC relaxation room (7%) to ameliorate dehydration. The health administration also got exhaust fans installed in each patient room and ICUs. This not only helped in heat reduction but also reduced the risk of suspended droplets in a closed space, by creating a negative pressure environment. Similar strategies have been supported by other researchers in the form of pre-cooling (drinking ice slurry), per-cooling (cooling vests), and post-cooling methods [9]. Heat stress and fluid loss have been perceived to be seriously restrictive when working in temperatures of 28 °C or more, which is quite common in India [10]. In our study, 149 respondents (49%) reported forced removal of PPE kit due to extreme heat or thirst on one or more occasions; 66 respondents (26%) reported forced removal of PPE kit due to the urge to void the bladder, and 185 respondents (73%) reported voiding bladders before donning to permit prolonged duration of PPE kit usage.

Of note, 216 (85.5%) respondents reported sufficient availability of PPE Kits, while 14 (5.5%) respondents reported a paucity of PPE kits on some occasions, forcing reuse, while another 23 (9%) declined to comment on this question. None of the respondents reported a severe or frequent shortage of PPE kits; this could be attributed to the fact that within 60 days into the pandemic, India had become the second-largest manufacturer of PPE kits in the world. Nevertheless, we should not forget that while PPE kits are a critical tool in the armamentarium of HCWs for combating the virus, they should be the last resort for the public. This valuable resource should be reserved for HCWs working in high-risk areas. Internationally, however, there have been reports of a shortage of PPE kits from the US, Russia, Canada, UK, Ireland, Australia, China, Pakistan, and a host of other countries [11-15]. A survey conducted across Europe and Australia has reported a shortage of PPE kits to the tune of 52% on at least one occasion and 30% incidence of reuse [16]. This was probably due to the fact that they had conducted the survey in the initial phase of the pandemic when it had just set in and the healthcare infrastructure was undergoing the process of upgradation in response to the disease onslaught. To address the shortage of supply chain, health administrators have resorted to increasing production, rationing, using the type of PPEs as per the level of protection required in particular hospital areas, do-it-yourself (DIY) measures, reuse after ultra-violet C germicidal irradiation (UVGI), gamma or X-ray irradiation methods of sterilisation without affecting the efficacy, usage of improvised surgical gowns that can be washed, autoclaved and augmented with disposable aprons, surgical caps, shoe covers, etc [6,17-18]. However, different sterilisation methods have various limitations, which can affect widespread use [19]. In our study, 103 respondents (41%) rationalised PPE kit usage by using disposable surgical gowns in low-risk areas along with N95 masks, surgical caps, shoe covers, gloves, and face shields. Isolation gowns/jumpsuits/hazmat suits were used as PPE kits only in high-risk areas. Some respondents also reported planning out their work in advance before donning to make effective use of the valuable kit-on time.

In our survey, 153 respondents (61%) did not report any size-related issues with PPE kits and reported that there was free availability of various sizes of or free-size PPE kits; 98 respondents (39%) reported issues with size while using PPE kits or any of its components. Size issue has been most commonly attributed to extremes of height, higher BMI, and facial hair [20].

In our study, 113 (45%) respondents said they used N95 masks only, while 140 respondents (55%) reported using both N95 masks and surgical masks simultaneously. The usage of simple three-ply surgical masks over the N95 mask was done possibly for two reasons. One reason was to make the N95 mask secure a tight fit on the face leaving no gaps between the rims of the mask and areas around the nose and mouth, while the other reason was to increase the life span and re-usability of the N95 mask. Of note, 122 respondents (48.3%) reported using single pairs of gloves while 110 respondents (43.5%) used double pairs of gloves, which reportedly helped in various stages of doffing. Also, 231 (91.3%) respondents reported using sanitizer on gloved hands while doffing at each stage. Double gloves have been reported to result in reduced dexterity in fine manual work [10].

One hundred sixty-one (64%) respondents reported getting formal training in donning, doffing, and other aspects of the usage of PPE kits. Knowledge augmentation and shortcomings in formal training were overcome by watching online videos (125 respondents, 49%), and taking part in online webinars (80 respondents, 32%). Active training in donning and doffing PPE has been shown to definitely boost the confidence level and reduce the risk of contamination and infection of HCWs [21]. Of note, 110 respondents (44%) reported PPE kits getting torn at one or more places on at least one occasion during doffing or donning. This is potentially a very serious problem associated with the use of PPE kit. Two hundred thirty-five respondents (93%) reported having a well designated separate area earmarked for donning and doffing; 18 respondents (7%) denied having a separate dedicated area for donning or doffing. One hundred twenty-two respondents (49%) reported that there was no one to help in donning or doffing, while 35 respondents (14%) reported that no help was required. To overcome this problem, 54 respondents (21%) reported donning and doffing in pairs and usage of a full-length mirror (18%) installed in respective donning and doffing areas. This has been termed the buddy system of doffing PPE kits [22]. Respondents also reported keeping pictorial steps of donning and doffing in respective areas. Some reported the usage of mobile phone cameras in donning areas where mirrors were not available.

**Figure 3:**
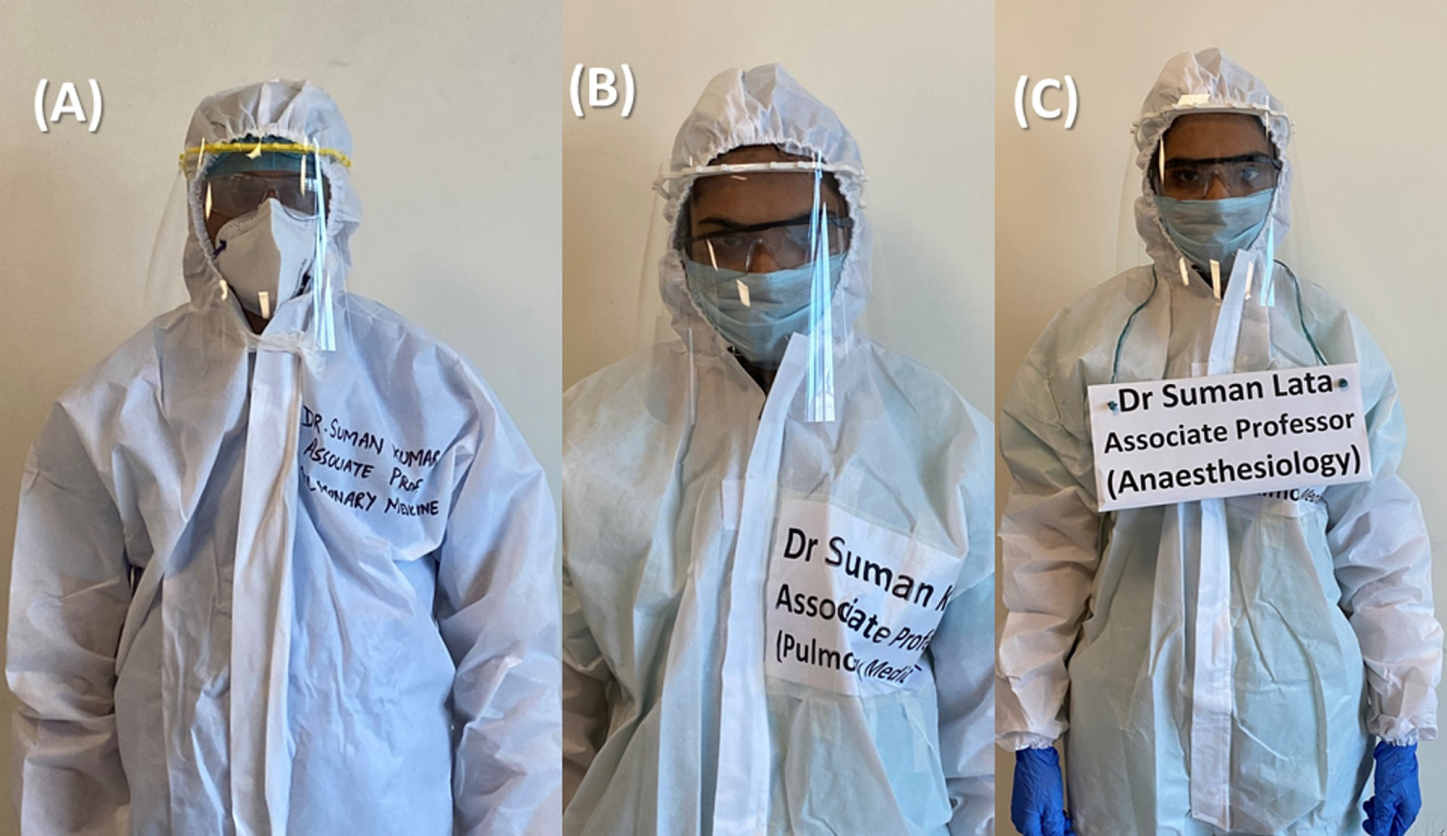
Evolution of methods to solve identification problems A: marking; B: labelling; C: placard (hypothetical names)

**Figure 4:**
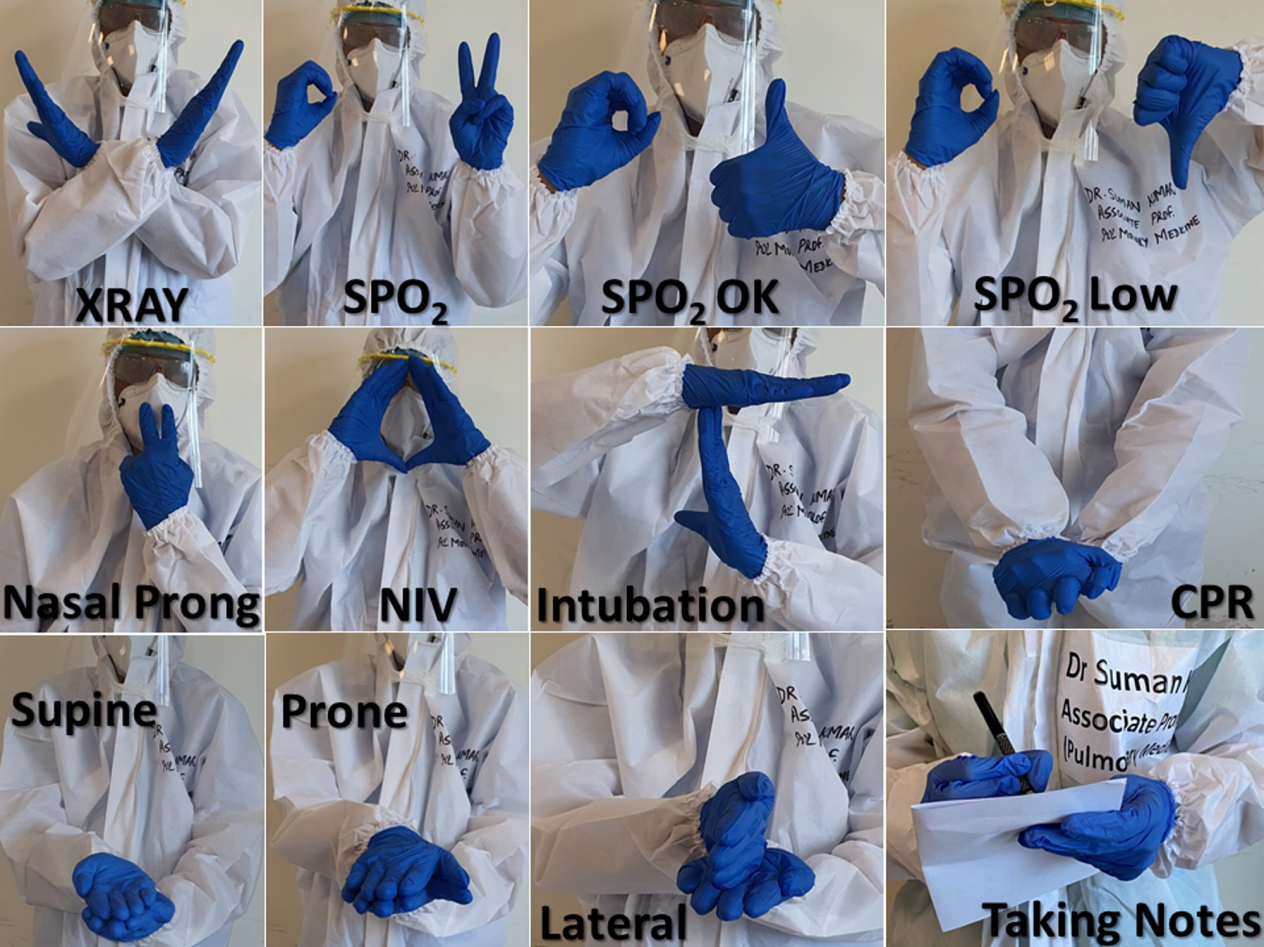
Sign language developed for ease of communication SPO_2_: oxygen saturation; NIV: non-invasive ventilation; CPR: cardiopulmonary resuscitation

One hundred forty respondents (55%) reported problems and confusion due to patients not recognising the staff in the PPE kit, which was overcome by writing the name and designation on PPE kits before donning, or by wearing disposable placards (Figure 3); 203 (80%) respondents reported facing communication issues with patients and colleagues after donning PPE kits, at least sometimes or frequently. This issue related to communication, mostly difficulty in hearing and understanding speech, has been corroborated by other studies, which also suggest a definitive direct effect on situational awareness [23]. This issue was addressed to some extent by devising sign language for commonly used phrases or directions (Figure 4).

Significantly, 64 respondents out of the 86 (74%) who carried out intubation reported facing difficulty in intubating patients with PPE kits on; 35 out of 40 respondents (88%) who gave CPR at some time reported facing difficulty in giving effective resuscitation with PPE kits on.

Other occasional problems reported were skin allergy/dermatitis caused by synthetic material of the PPE kit, face shields impinging onto the neck during intubation, and nasal pain, pain at the root of the pinna, and slipperiness of shoe covers. Contact dermatitis/eczema caused by the material of the PPE components has also been reported from elsewhere, especially in high-friction and perspiration areas such as the chin, jaw, ears, eyelids, and arm-pits [24]. Two respondents admitted to developing a fear of certain areas of the body remaining exposed while using PPE kits. Three respondents also admitted to trying to stay calm, meditating, or praying to God when they had the urge to remove the kit for one or other reasons.

There are certain problems reported in the literature from other countries that none of our respondents have enumerated. For example, studies have reported dissatisfaction with work, a statistically significant drop in oxygen saturation, and an increase in pulse rate after wearing PPE for four hours as compared to baseline. Another finding has been that most of the participants tended to adjust their N95 masks intermittently due to breathing issues, which raises the risk of self-contamination [25]. There are also certain solutions suggested in various studies, which include reduction of non-urgent surgical procedures, refinement in the use of PPEs from disposable to re-usable ones, and replacement of aerosol-generating medical procedures like general anaesthesia with regional anaesthesia wherever possible [26]. Another possible solution for the shortage is the use of low-cost, effective reusable customized PPE made of water-impervious warp and weft polyester fabric 190 threads, consisting of full inner coverall with hood, outer gown, shoe cover, and plastic face shield, which could be disinfected in 1% hypochlorite solution for 20 minutes [27]. There has been an interesting report of a child getting frightened at seeing someone in a PPE kit, and tweaking the surface of the kit with cartoon stickers worked wonders, making the PPE suit more child-friendly [28].

The strengths of our study include surveying all strata of HCWs who had used PPE kits. Disclosing the shorter average response time of three minutes for the questionnaire, assurance of the confidentiality of data, and making subject/institution name submission optional helped in increasing the acceptability of the survey questionnaire towards this sensitive topic, which has been under much media glare.

## Conclusions

We believe that this first-of-its-kind, non-funded survey among HCWs, conducted in a country that has experienced some of the biggest burdens due to the COVID-19 pandemic, should serve as a guide to health administrators as well as other HCWs in adopting ways and means to ameliorate the problems encountered in the use of PPE kits.
